# Supplementary material for: A Likelihood Approach for Real-Time Calibration of Stochastic Compartmental Epidemic Models
Source: PLoS Comput Biol. 2017 Jan 17;13(1):e1005257. doi: 10.1371/journal.pcbi.1005257 (PMC5240920; doi:10.1371/journal.pcbi.1005257)
Supplement: S1 File — (TAR.GZ) [file pcbi.1005257.s014.tar.gz › HSPH_Online-SI-Revision/PF08/n100-severe/n100-severe_PF_table0.pdf]

1	R0	{1.65278, 9.06793}	{2.18753, 20.}	{{2.14624, 20.}, {2.26394, 20.}}	{32.3546, 120.558}	inf	9005.	19 898.8	{18 904.1, 20 462.5}	120.975	0.34417	newly	6768.	7049.44	{6702., 7268.}	4.6906	0.0198452	Reff	0.958398	1.12145	{1.10769, 1.13469}	17.0131	ar	66 342.	96 134.4	{95 297., 97 441.}	44.9073
2	R0	{1.4321, 3.80644}	{3., 20.}	{{3., 20.}, {3., 20.}}	{109.483, 425.425}	inf	4783.	32 272.6	{32 104.9, 32 441.9}	574.735	0.82913	newly	7700.	11 135.2	{11 031., 11 232.}	44.6123	0.160196	Reff	0.875729	1.03187	{1.01277, 1.0639}	17.8296	ar	54 217.	100 478.	{100 117., 100 827.}	85.3263
3	R0	{1.68388, 4.76028}	{3., 20.}	{{3., 20.}, {3., 20.}}	{78.16, 320.143}	inf	8327.	32 866.	{32 657.4, 33 036.3}	294.692	0.596255	newly	10 200.	11 510.8	{11 378., 11 657.}	12.8514	0.0524887	Reff	0.78489	1.12888	{1.12584, 1.13174}	43.8266	ar	68 299.	102 564.	{102 169., 102 916.}	50.1691
4	R0	{1.66014, 6.95383}	{2.9015, 20.}	{{2.85653, 20.}, {2.9436, 20.}}	{74.7743, 187.611}	inf	9062.	29 845.	{29 250.8, 30 213.9}	229.342	0.517585	newly	8732.	10 361.3	{10 170., 10 513.}	18.6588	0.0742531	Reff	0.943906	1.05846	{1.03278, 1.08912}	12.1367	ar	66 820.	98 098.7	{97 563., 98 557.}	46.8104
5	R0	{1.69984, 11.2038}	{2.2554, 20.}	{{2.18574, 20.}, {2.31564, 20.}}	{32.6832, 78.5109}	inf	9747.	19 227.6	{18 481.8, 20 468.6}	97.2666	0.294765	newly	5980.	6700.12	{6417., 7139.}	12.0421	0.0490852	Reff	0.958166	1.02953	{0.993147, 1.0531}	7.44759	ar	68 462.	96 572.7	{95 375., 97 504.}	41.0603
6	R0	{1.65876, 6.66217}	{2.80036, 20.}	{{2.75832, 20.}, {2.82541, 20.}}	{68.8223, 200.202}	inf	9053.	27 482.5	{26 881.9, 27 919.4}	203.573	0.482219	newly	9118.	9824.98	{9585., 10 026.}	7.75367	0.0323745	Reff	1.0042	1.23095	{1.20219, 1.25454}	22.5809	ar	67 105.	96 909.4	{96 231., 97 696.}	44.4146
7	R0	{1.69966, 5.89996}	{3., 20.}	{{3., 20.}, {3., 20.}}	{76.5059, 238.985}	inf	10 219.	31 456.7	{31 275.1, 31 656.1}	207.826	0.4883	newly	11 408.	11 108.5	{10 978., 11 234.}	2.62526	0.0115683	Reff	0.93415	1.17203	{1.14758, 1.19566}	25.4644	ar	69 396.	99 531.8	{99 288., 99 827.}	43.4259
8	R0	{1.53428, 6.3734}	{2.19436, 17.6244}	{{2.17463, 16.9159}, {2.2128, 18.0826}}	{43.0222, 176.531}	inf	6765.	19 439.3	{18 989.6, 19 830.2}	187.352	0.45833	newly	7091.	7886.	{7727., 8094.}	11.2114	0.046062	Reff	0.973639	1.17308	{1.16003, 1.19197}	20.4842	ar	59 855.	91 078.3	{90 313., 91 784.}	52.1649
9	R0	{1.58939, 3.48608}	{3., 20.}	{{3., 20.}, {3., 20.}}	{88.7517, 473.71}	inf	7643.	24 271.5	{24 179.2, 24 352.1}	217.565	0.501831	newly	13 814.	9517.52	{9386., 9634.}	31.1024	0.161814	Reff	0.959229	1.79792	{1.79474, 1.80134}	87.4343	ar	62 575.	97 891.1	{97 680., 98 083.}	56.4381
10	R0	{1.58528, 5.5593}	{1.97401, 20.}	{{1.97298, 20.}, {1.97728, 20.}}	{24.5215, 259.758}	inf	7290.	12 150.	{12 135.9, 12 174.5}	66.6665	0.221846	newly	8668.	4542.87	{4444., 4611.}	47.5903	0.280626	Reff	0.933207	1.4169	{1.41566, 1.41983}	51.8312	ar	63 039.	84 471.3	{84 150., 84 774.}	33.9985
11	R0	{1.58305, 4.62721}	{3., 20.}	{{3., 20.}, {3., 20.}}	{89.5076, 332.226}	inf	7294.	32 828.6	{32 582.1, 33 005.7}	350.077	0.65328	newly	9915.	11 472.8	{11 295., 11 586.}	15.7111	0.0633559	Reff	0.910428	1.10652	{1.08094, 1.13557}	21.539	ar	63 222.	101 547.	{101 166., 101 850.}	60.619
12	R0	{1.70234, 9.34626}	{2.00477, 20.}	{{1.99906, 20.}, {2.00907, 20.}}	{17.7658, 113.989}	inf	9412.	14 563.6	{14 434.6, 14 646.1}	54.7345	0.189581	newly	6925.	5315.7	{5230., 5408.}	23.239	0.114894	Reff	0.976973	1.28653	{1.28452, 1.28923}	31.6854	ar	68 213.	86 113.4	{85 817., 86 437.}	26.2419
13	R0	{1.68556, 9.74954}	{2.22271, 20.}	{{2.15167, 20.}, {2.32098, 20.}}	{31.8677, 105.138}	inf	9667.	19 910.9	{19 155.5, 20 716.3}	105.967	0.313612	newly	6771.	6977.01	{6655., 7250.}	3.72781	0.0158312	Reff	0.938014	1.06158	{1.03353, 1.08798}	13.1735	ar	68 426.	95 352.3	{93 725., 97 261.}	39.351
14	R0	{1.59876, 9.78897}	{2.2075, 20.}	{{2.16552, 20.}, {2.24792, 20.}}	{38.0758, 104.312}	inf	7640.	17 249.5	{16 712.6, 17 675.7}	125.779	0.353566	newly	5391.	6172.7	{5968., 6350.}	14.5001	0.0586708	Reff	1.0474	1.17502	{1.15705, 1.18012}	12.1845	ar	63 553.	93 755.1	{92 693., 94 588.}	47.5228
15	R0	{1.43577, 5.12819}	{2.21507, 20.}	{{2.20386, 20.}, {2.22099, 20.}}	{54.2774, 290.001}	inf	5339.	18 415.5	{18 161.8, 18 550.8}	244.924	0.537711	newly	6966.	6767.35	{6655., 6870.}	2.86664	0.0126646	Reff	0.97842	1.33548	{1.33293, 1.33934}	36.4932	ar	53 259.	92 292.2	{91 872., 92 742.}	73.2894
16	R0	{1.58866, 8.73191}	{2.1396, 20.}	{{2.13015, 20.}, {2.14853, 20.}}	{34.6797, 129.045}	inf	7934.	18 015.7	{17 881.4, 18 190.7}	127.069	0.356148	newly	6295.	6581.11	{6465., 6696.}	4.54504	0.019265	Reff	1.05624	1.29631	{1.29194, 1.30054}	22.7296	ar	64 349.	93 388.3	{92 683., 93 899.}	45.1278
17	R0	{1.43012, 3.8163}	{2.99153, 20.}	{{2.96714, 20.}, {3., 20.}}	{109.18, 424.068}	inf	5239.	29 547.7	{28 934.6, 30 090.9}	463.996	0.751208	newly	8949.	10 753.1	{10 493., 10 997.}	20.1596	0.0796844	Reff	0.975428	1.35687	{1.33858, 1.37871}	39.1048	ar	53 353.	99 804.	{99 254., 100 492.}	87.0635
18	R0	{1.47965, 5.16472}	{2.27264, 15.6362}	{{2.0709, 13.6617}, {2.70998, 20.}}	{53.5929, 202.75}	inf	5515.	21 602.6	{18 353.9, 28 767.6}	291.706	0.585994	newly	7086.	9836.92	{9394., 10 287.}	38.8219	0.142174	Reff	0.947154	1.1805	{1.14271, 1.22731}	24.6365	ar	56 086.	95 389.8	{91 919., 102 103.}	70.0778
19	R0	{1.57191, 5.2314}	{2.90382, 20.}	{{2.85233, 20.}, {2.93185, 20.}}	{84.732, 282.307}	inf	6751.	29 795.5	{28 890.1, 30 338.}	341.35	0.644706	newly	7954.	10 154.7	{9858., 10 393.}	27.6675	0.105988	Reff	0.829937	0.936894	{0.920511, 0.958734}	12.8874	ar	63 119.	99 015.	{98 346., 99 884.}	56.8703
20	R0	{1.54435, 5.32911}	{2.32013, 20.}	{{2.31507, 20.}, {2.32342, 20.}}	{50.2333, 275.297}	inf	6968.	20 259.9	{20 125.6, 20 345.}	190.757	0.463526	newly	8356.	7412.32	{7309., 7508.}	11.2934	0.0520702	Reff	0.898704	1.32787	{1.32616, 1.32969}	47.7534	ar	61 870.	94 501.9	{94 193., 94 817.}	52.7427
21	R0	{1.57932, 4.90031}	{3., 20.}	{{3., 20.}, {3., 20.}}	{89.9552, 308.137}	inf	8141.	31 262.3	{31 093.4, 31 411.1}	284.01	0.584339	newly	10 527.	11 068.2	{10 936., 11 188.}	5.14097	0.0217536	Reff	0.930156	1.18167	{1.16709, 1.20613}	27.0399	ar	63 595.	99 435.2	{99 070., 99 670.}	56.3569
22	R0	{1.47044, 4.07694}	{2.18605, 17.4271}	{{2.12545, 16.7152}, {2.2082, 17.6926}}	{48.6664, 327.456}	inf	5758.	14 865.3	{14 154.4, 15 147.5}	158.168	0.411761	newly	9422.	6552.69	{6419., 6668.}	30.4533	0.15777	Reff	0.991724	1.52626	{1.50039, 1.53648}	53.8994	ar	55 895.	89 485.5	{88 080., 90 176.}	60.0957
23	R0	{1.64997, 3.34546}	{3., 20.}	{{3., 20.}, {3., 20.}}	{81.8215, 497.825}	inf	6829.	31 277.6	{31 127.5, 31 428.7}	358.011	0.660873	newly	10 882.	11 441.3	{11 311., 11 570.}	5.13931	0.021747	Reff	0.75727	1.39673	{1.39238, 1.40009}	84.4422	ar	66 404.	104 413.	{103 880., 104 873.}	57.2387
24	R0	{1.67405, 4.50254}	{3., 20.}	{{3., 20.}, {3., 20.}}	{79.2061, 344.194}	inf	9359.	29 626.	{29 438.1, 29 777.8}	216.551	0.500441	newly	12 907.	10 972.8	{10 838., 11 107.}	14.9854	0.0705235	Reff	0.908708	1.47361	{1.47077, 1.47693}	62.1659	ar	68 017.	100 833.	{100 500., 101 151.}	48.247
25	R0	{1.50663, 3.1286}	{3., 20.}	{{3., 20.}, {3., 20.}}	{99.1199, 539.264}	inf	5849.	29 297.6	{28 831., 29 544.9}	400.9	0.69973	newly	11 133.	10 922.	{10 709., 11 090.}	1.92248	0.00846595	Reff	0.89994	1.50668	{1.49362, 1.51424}	67.4201	ar	58 823.	101 667.	{101 217., 102 099.}	72.8358
26	R0	{1.69822, 6.34992}	{2.17707, 17.6733}	{{2.17443, 17.6483}, {2.17877, 17.7102}}	{28.1972, 178.323}	inf	9988.	16 620.8	{16 560.7, 16 667.5}	66.4073	0.221171	newly	10 139.	7018.43	{6908., 7103.}	30.7779	0.159784	Reff	0.906629	1.38446	{1.38246, 1.38676}	52.7043	ar	69 816.	89 125.4	{88 876., 89 355.}	27.6576
27	R0	{1.55869, 4.92988}	{2.80477, 20.}	{{2.78952, 20.}, {2.81493, 20.}}	{79.9437, 305.689}	inf	7340.	28 287.6	{28 042.4, 28 495.5}	285.39	0.585892	newly	9500.	10 087.1	{9964., 10 215.}	6.18	0.0260217	Reff	0.908124	1.22076	{1.21378, 1.22797}	34.427	ar	62 344.	99 014.2	{98 753., 99 270.}	58.8191
28	R0	{1.46085, 6.06899}	{1.98177, 14.157}	{{1.89712, 12.95}, {2.06845, 15.2592}}	{35.659, 133.268}	inf	5497.	16 602.4	{14 594.2, 18 627.6}	202.026	0.477649	newly	5894.	8298.72	{7823., 8768.}	40.7995	0.148154	Reff	0.925463	1.10576	{1.06921, 1.14417}	19.4819	ar	55 484.	90 884.6	{89 049., 93 049.}	63.8033
29	R0	{1.63017, 6.25164}	{2.93097, 20.}	{{2.88357, 20.}, {2.96336, 20.}}	{79.7953, 219.916}	inf	8283.	30 167.2	{29 380.5, 30 707.}	264.207	0.561287	newly	8506.	10 347.4	{10 060., 10 548.}	21.648	0.0850441	Reff	0.896577	0.983789	{0.953265, 1.01226}	9.72723	ar	66 231.	98 491.7	{97 692., 99 380.}	48.7094
30	R0	{1.45173, 5.68165}	{2.18863, 20.}	{{2.18071, 20.}, {2.19377, 20.}}	{50.7603, 252.01}	inf	5301.	15 325.4	{15 133.5, 15 454.3}	189.103	0.461042	newly	6273.	5599.85	{5498., 5676.}	10.7309	0.0493329	Reff	0.953496	1.29504	{1.29312, 1.29749}	35.8205	ar	55 280.	90 639.7	{90 177., 90 996.}	63.9647
31	R0	{1.55808, 2.77316}	{3., 20.}	{{3., 20.}, {3., 20.}}	{92.5447, 621.199}	inf	4924.	29 563.4	{29 416.4, 29 682.3}	500.394	0.778434	newly	9080.	10 980.6	{10 868., 11 115.}	20.9319	0.0825235	Reff	0.782265	1.48331	{1.48091, 1.48716}	89.6177	ar	60 940.	101 124.	{100 830., 101 407.}	65.9406
32	R0	{1.56797, 3.90327}	{3., 20.}	{{3., 20.}, {3., 20.}}	{91.3302, 412.391}	inf	6996.	29 865.7	{29 741.4, 29 996.1}	326.897	0.630321	newly	11 191.	10 934.8	{10 812., 11 047.}	2.28934	0.0100758	Reff	0.933679	1.39624	{1.39289, 1.39923}	49.5415	ar	62 228.	99 780.6	{99 564., 100 055.}	60.3468
33	R0	{1.70027, 5.43261}	{3., 20.}	{{3., 20.}, {3., 20.}}	{76.4426, 268.147}	inf	8804.	33 318.5	{33 142.1, 33 503.9}	278.447	0.578002	newly	10 094.	11 486.3	{11 355., 11 609.}	13.7931	0.056101	Reff	0.817558	1.02697	{1.02268, 1.03257}	25.6148	ar	68 798.	104 371.	{103 924., 104 793.}	51.7061
34	R0	{1.61668, 6.86192}	{1.94081, 20.}	{{1.93674, 20.}, {1.94449, 20.}}	{20.0491, 191.464}	inf	8157.	11 679.9	{11 610.2, 11 747.5}	43.1882	0.155902	newly	7767.	4361.24	{4276., 4430.}	43.8491	0.250683	Reff	0.928411	1.39827	{1.39578, 1.3994}	50.609	ar	64 693.	83 182.	{82 781., 83 532.}	28.5796
35	R0	{1.55955, 3.45071}	{3., 20.}	{{3., 20.}, {3., 20.}}	{92.3632, 479.591}	inf	6759.	28 552.7	{28 402.5, 28 681.8}	322.439	0.625762	newly	11 360.	10 783.5	{10 641., 10 918.}	5.07518	0.0226409	Reff	0.876124	1.5864	{1.58219, 1.58911}	81.0699	ar	61 783.	103 464.	{102 956., 103 977.}	67.4629
36	R0	{1.65068, 4.91307}	{2.99667, 20.}	{{2.99126, 20.}, {3., 20.}}	{81.5418, 307.077}	inf	9077.	28 820.2	{28 696.1, 28 910.6}	217.508	0.501752	newly	12 338.	10 639.6	{10 511., 10 751.}	13.7654	0.0643381	Reff	0.997374	1.44721	{1.44234, 1.45431}	45.1018	ar	66 453.	99 547.3	{99 372., 99 744.}	49.801
37	R0	{1.46131, 3.06714}	{3., 20.}	{{3., 20.}, {3., 20.}}	{105.295, 552.073}	inf	5039.	31 341.	{31 215.3, 31 441.8}	521.969	0.793768	newly	9251.	11 258.9	{11 172., 11 378.}	21.7049	0.0852961	Reff	0.841612	1.281	{1.27864, 1.28403}	52.2079	ar	56 452.	102 007.	{101 646., 102 305.}	80.6965
38	R0	{1.71034, 5.2546}	{3., 20.}	{{3., 20.}, {3., 20.}}	{75.4037, 280.619}	inf	9467.	33 132.6	{32 836.9, 33 308.5}	249.98	0.544037	newly	11 210.	11 532.2	{11 393., 11 645.}	2.87395	0.0122888	Reff	0.857787	1.0873	{1.08336, 1.09159}	26.756	ar	69 816.	101 881.	{101 294., 102 315.}	45.928
39	R0	{1.43565, 5.57989}	{2.02501, 14.3894}	{{1.90258, 12.7704}, {2.41413, 20.}}	{41.0516, 157.88}	inf	5165.	17 134.7	{14 925.3, 24 065.5}	231.747	0.514618	newly	6041.	8320.93	{7958., 8652.}	37.7409	0.138853	Reff	0.90825	1.06265	{1.03109, 1.09091}	17.0003	ar	54 407.	87 462.3	{84 251., 96 009.}	60.7556
40	R0	{1.40327, 4.00764}	{2.63393, 18.4801}	{{2.59874, 17.9497}, {2.69702, 19.1191}}	{87.6997, 361.122}	inf	4452.	26 719.4	{26 303., 27 773.4}	500.166	0.777337	newly	7170.	10 417.8	{10 098., 10 687.}	45.2965	0.162161	Reff	0.942464	1.25957	{1.21717, 1.29782}	33.6462	ar	51 976.	100 995.	{99 932., 101 988.}	94.3108
41	R0	{1.63612, 4.26741}	{3., 20.}	{{3., 20.}, {3., 20.}}	{83.3606, 368.668}	inf	7141.	32 606.1	{32 465.8, 32 730.2}	356.605	0.659538	newly	9905.	11 568.8	{11 433., 11 696.}	16.7977	0.0674193	Reff	0.840131	1.20482	{1.20264, 1.20891}	43.409	ar	65 029.	103 611.	{103 279., 103 913.}	59.3307
42	R0	{1.57426, 6.50271}	{2.20896, 20.}	{{2.20442, 20.}, {2.21321, 20.}}	{40.3176, 207.564}	inf	7623.	18 913.1	{18 801., 19 021.6}	148.106	0.394632	newly	7814.	6931.72	{6818., 7040.}	11.291	0.0520684	Reff	0.952711	1.32435	{1.32228, 1.32677}	39.0086	ar	62 522.	94 812.8	{94 351., 95 258.}	51.6471
43	R0	{1.37818, 5.37636}	{2.18246, 20.}	{{2.1435, 20.}, {2.21643, 20.}}	{58.3585, 271.999}	inf	4296.	19 278.7	{18 695.8, 19 966.3}	348.76	0.65188	newly	5382.	6945.85	{6758., 7175.}	29.057	0.110649	Reff	0.94794	1.22125	{1.20483, 1.23217}	28.8321	ar	50 098.	94 016.	{93 365., 94 690.}	87.6641
44	R0	{1.66606, 4.92641}	{3., 20.}	{{3., 20.}, {3., 20.}}	{80.0655, 305.975}	inf	9374.	29 602.5	{29 436.3, 29 752.8}	215.794	0.4994	newly	12 427.	10 904.9	{10 760., 11 011.}	12.2487	0.0567636	Reff	0.958134	1.42965	{1.4246, 1.4375}	49.2117	ar	67 066.	99 831.6	{99 619., 100 077.}	48.8558
45	R0	{1.623, 5.9449}	{2.88155, 20.}	{{2.83744, 20.}, {2.93406, 20.}}	{77.5448, 236.423}	inf	8468.	28 372.	{27 837.2, 29 055.8}	235.049	0.525058	newly	9497.	10 155.7	{9986., 10 388.}	6.93587	0.0290723	Reff	0.985843	1.24716	{1.22812, 1.26403}	26.5074	ar	64 554.	99 366.3	{97 973., 100 044.}	53.9275
46	R0	{1.65235, 5.51234}	{2.14377, 17.0087}	{{2.14094, 16.9918}, {2.1464, 17.0495}}	{29.7406, 208.557}	inf	8716.	13 940.2	{13 887.4, 13 988.4}	59.9378	0.203949	newly	10 647.	6298.45	{6195., 6406.}	40.843	0.228028	Reff	0.988634	1.51228	{1.51067, 1.51384}	52.9663	ar	66 509.	87 441.7	{87 153., 87 766.}	31.4735
47	R0	{1.62138, 8.82208}	{2.2087, 19.7952}	{{2.17413, 20.}, {2.24769, 20.}}	{36.2233, 124.383}	inf	8399.	20 274.1	{19 642.5, 20 954.4}	141.387	0.382254	newly	6515.	7154.8	{6895., 7358.}	9.82041	0.0405565	Reff	0.936704	1.05081	{1.02648, 1.07227}	12.1816	ar	65 598.	92 724.1	{91 547., 93 783.}	41.3521
48	R0	{1.64073, 6.69261}	{2.87065, 19.8941}	{{2.80622, 20.}, {2.93606, 20.}}	{74.9619, 197.255}	inf	8727.	29 079.7	{28 491.8, 29 844.}	233.215	0.522289	newly	8793.	10 240.	{10 028., 10 435.}	16.4563	0.06608	Reff	0.968375	1.11493	{1.08396, 1.15256}	15.1344	ar	66 438.	98 078.3	{97 332., 99 142.}	47.6238
49	R0	{1.65556, 3.796}	{3., 20.}	{{3., 20.}, {3., 20.}}	{81.2076, 426.87}	inf	8332.	28 296.	{28 164.6, 28 419.2}	239.606	0.530972	newly	12 770.	10 809.4	{10 685., 10 924.}	15.3532	0.0724064	Reff	0.845809	1.65565	{1.65127, 1.65884}	95.748	ar	66 575.	103 463.	{103 018., 103 865.}	55.4089
50	R0	{1.56828, 3.65713}	{3., 20.}	{{3., 20.}, {3., 20.}}	{91.2924, 446.877}	inf	6216.	32 650.3	{32 456.3, 32 788.5}	425.261	0.720372	newly	9733.	11 640.4	{11 498., 11 778.}	19.597	0.0777034	Reff	0.821841	1.23905	{1.23649, 1.2449}	50.765	ar	62 761.	104 229.	{103 812., 104 663.}	66.0724
